# Green Tea and Other Tea Polyphenols: Effects on Sebum Production and Acne Vulgaris

**DOI:** 10.3390/antiox6010002

**Published:** 2016-12-29

**Authors:** Suzana Saric, Manisha Notay, Raja K. Sivamani

**Affiliations:** 1 School of Medicine, University of California—Davis, Sacramento, CA 95817, USA; ssaric@ucdavis.edu; 2 Department of Dermatology, University of California—Davis, Sacramento, CA 95816, USA; mnotay@ucdavis.edu

**Keywords:** tea, polyphenol, EGCG, catechin, sebum, acne vulgaris

## Abstract

Polyphenols are antioxidant molecules found in many foods including nuts, fruits, vegetables, chocolate, wine, and tea. Polyphenols have antimicrobial, anti-inflammatory, and antineoplastic properties. Recent studies suggest that tea polyphenols may be used for reducing sebum production in the skin and for treatment of acne vulgaris. This review examines the evidence for use of topically and orally ingested tea polyphenols against sebum production and for acne treatment and prevention. The PubMed database was searched for studies on tea polyphenols, sebum secretion, and acne vulgaris. Of the 59 studies found, eight met the inclusion criteria. Two studies evaluated tea polyphenol effects on sebum production; six studies examined tea polyphenol effects on acne vulgaris. Seven studies evaluated topical tea polyphenols; one study examined systemic tea polyphenols. None of the studies evaluated both topical and systemic tea polyphenols. Tea polyphenol sources included green tea (six studies) and tea, type not specified (two studies). Overall, there is some evidence that tea polyphenols in topical formulation may be beneficial in reducing sebum secretion and in treatment of acne. Research studies of high quality and with large sample sizes are needed to assess the efficacy of tea polyphenols in topical and oral prevention of acne vulgaris and lipid synthesis by the sebaceous glands.

## 1. Introduction

Polyphenols are naturally occurring compounds found in plants and common foods such as nuts, fruits, vegetables, chocolate, wine, and tea. After ingestion, polyphenols are absorbed in the intestinal epithelium [1] and have been shown to reach systemic circulation [2,3,4,5]. Polyphenols are antioxidants and play a role in preventing oxidative damage caused by the reactive oxygen species (ROS) [6]. They possess anti-inflammatory [7] and anti-carcinogenic properties and may aid in the prevention of cardiovascular disease [8], as well as protect skin from ultraviolet radiation (UVR) [9,10,11].

**Figure 1:**
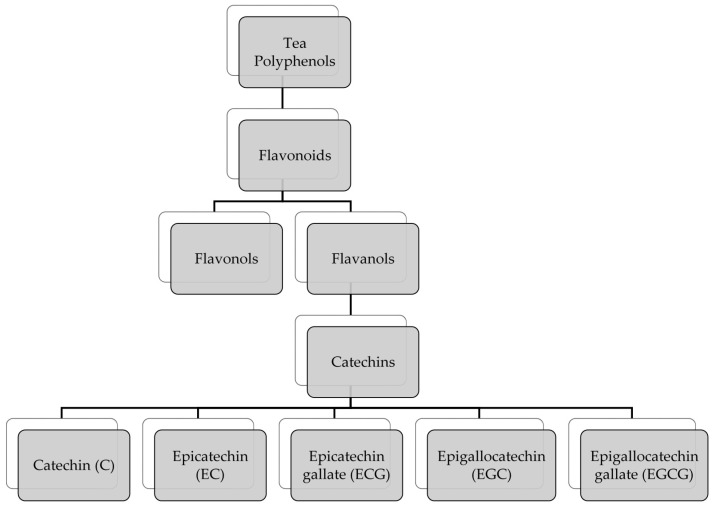
Major components of the tea polyphenol family.

**Figure 2:**
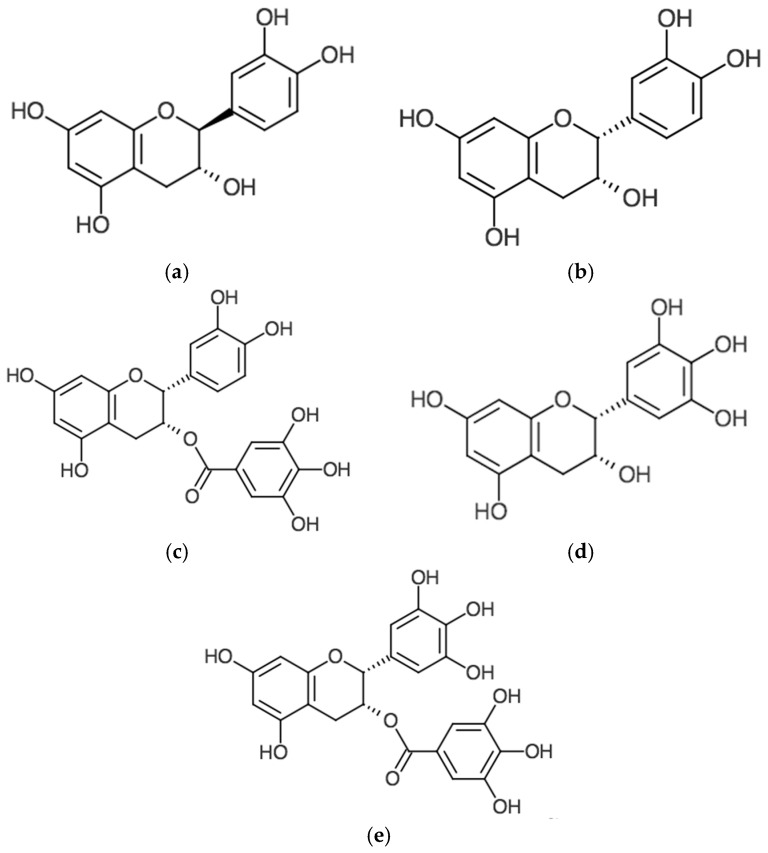
Chemical structures of major tea polyphenols. (**a**) (+)-Catechin (C); (**b**) (−)-Epicatechin (EC); (**c**) (−)-Epicatechin gallate (ECG); (**d**) (−)-Epigallocatechin (EGC); (**e**) (−)-Epigallocatechin gallate (EGCG).

Tea is the second most consumed beverage worldwide and an important source of plant polyphenols in the human diet [12]. *Camellia sinensis* is the plant that gives rise to a variety of teas depending on specific processing of the plant [13]. Green tea is produced from fresh leaves in such a way that prevents oxidation of polyphenolic components (mainly catechins), oolong tea polyphenols are partially oxidized, while polyphenols in black tea undergo a high degree of oxidation [13]. Components of green tea beverage measured as weight percentage of extract solids include 30%–42% catechins, 5%–10% flavonols, and 2%–4% other flavonoids [13]. Catechins are divided into catechin (C), (−)-epicatechin (EC), (−)-epicatechin gallate (ECG), (−)-epigallocatechin (EGC), and (−)-epigallocatechin-3-gallate (EGCG) (Figure 1 and Figure 2). EGCG is the most abundant catechin and has been shown to have beneficial health effects on skin [12].

Sebaceous glands are found throughout the human body, especially on the face and scalp, and they produce sebum—a mixture of lipids [14]. Lipids are produced by other glands in the body but the two most specific lipids to sebaceous glands are squalene and wax esters [14]. Sebum secretion is associated with hormonal activity in the human body [15] and excess sebum production is implicated in the development of skin disorders, such as acne vulgaris. Acne is not a life threatening condition but it can leave permanent scaring on the face and cause physical and psychological morbidities [16]. Acne may be caused by multiple mechanisms including (a) increased sebum production by the sebaceous glands; (b) altered keratinization within pilosebaceous follicles; (c) proliferation of bacteria *Propionibacterium acnes (P. acnes)*; and (d) inflammation around pilosebaceous follicles [16,17].

Recent evidence suggests that there is increased phosphoinositide 3-kinase-Akt-mammalian target of rapamycin complex 1 (PI3K-Akt-mTORC1) signaling in the skin of patients with acne vulgaris [18,19,20] and that increased PI3K-Akt-mTORC1 signaling induces sebum production [21]. The green tea-derived plant sterols have shown a therapeutic role in the treatment of acne vulgaris [22]. Im et al., 2012 provided experimental evidence that EGCG treatment of insulin growth factor (IGF-1) stimulated SZ95 sebocytes decreased mTOR phosphorylation and thus mTORC1 activity [23]. Additionally, Van Aller et al., 2011 [24] provided evidence that EGCG is an ATP-competitive inhibitor of both PI3K and mTOR. Molecular docking studies showed that EGCG binds well to the PI3K domain active site, agreeing with the findings that EGCG competes for ATP binding. The mTOR kinase also belongs to the family of PI3K related kinases. Increased insulin-IGF-1 signaling in acne vulgaris activates PI3K-Akt-mTORC1 signaling cascade and plays a key role in the Western-diet induced acne [25]. Natural mTORC1 inhibitors, such as green tea polyphenols, attenuate enhanced mTORC1 signaling [26]. When compared with other types of tea, green tea has the highest polyphenolic content, with EGCG and EGC components being the most abundant [27].

There are multiple acne grading systems but there is no gold standard [28] that is consistently used in clinical practice or research trials. Lesion counting has been validated as a reliable measure for acne [29] and requires counting the number of comedones, papules, pustules, and nodules on the face [30]. The global assessment scale classifies acne into one of five categories based on acne severity (very mild, mild, moderate, severe, and very severe) [30]. In the Leeds technique, a subject’s acne is compared to a photographic manual and a score from 1 (mild) to 10 (very severe) is assigned [30]. The Global Acne Grading System (GAGS) was developed in 1997 in an effort to improve accuracy, minimize inter- and intra-rater variability, and allow for quicker assessments [30].

Due to the complex nature of acne vulgaris, most treatments have only modest efficacy [31]. Systemic therapies that influence sebum production include estrogens, anti-androgens (i.e., spironolactone), retinoid (isotretinoin), and oral contraceptives [32]. All these medications predispose individuals to potentially serious side effects, thus there is a need for more natural therapies with minimal side effects. Research suggests that tea polyphenols may be promising in the management of acne vulgaris by reducing sebum production in the skin and acting as an anti-inflammatory [7] and antimicrobial agents [33]. In an in vitro study, Yoon et al., 2013 [31] showed that EGCG decreases lipogenisis in sebocytes, inhibits growth of *P. acnes* and reduces inflammation caused by *P. acnes*. The aim of this review is to examine recent clinical trials on tea polyphenols and their effects on sebum production and acne vulgaris management.

## 2. Materials and Methods

### 2.1. Search Strategy

On 4 August 2016 the PubMed database was searched for studies that investigated tea polyphenols, sebum production, and acne. No limits were placed on the publication date. The search combined keywords “tea”, “green tea”, “EGCG”, “polyphenol”, “catechin”, “sebum” and “acne”. No filters were selected.

### 2.2. Selection of Studies

Records were screened by title and/or abstract to exclude studies that did not contribute to answering the question in this review. Inclusion criteria: (1) published in English; (2) intervention included a tea polyphenol; (3) human study. Exclusion criteria: (1) in vitro studies; (2) animal studies; (3) review articles.

### 2.3. Data Extraction

Data was extracted from selected studies (Table A1) as follows: (1) tea; (2) polyphenol subtype and content; (3) therapy (topical or systemic; dosage); (4) comparison (or control/placebo); (5) subjects (*n*, age); (6) study design, duration; (7) major outcome measures; (8) major results; (9) adverse events; (10) reference.

## 3. Results

Our search yielded a total of 59 studies. Titles and/or abstracts were screened for inclusion and exclusion criteria. Eight articles met the criteria and were subsequently analyzed. Two studies examined the effects of tea polyphenols on sebum production, while six studies investigated the effects of tea polyphenols on acne vulgaris. Seven studies examined the application of topical tea polyphenols and one study examined systemic tea polyphenols. None of the studies examined both topical and systemic tea polyphenols. Tea polyphenol sources included green tea in six studies, while in two studies the tea type was not specified.

### 3.1. Sebum Production Studies

In a single-blinded, placebo controlled monocentric study by Mahmood et al., 2013 [15] conducted in Pakistan, 22 non-smoker, healthy men aged 22–28 years-old were enrolled to investigate the effects of green tea and lotus extract topicals on facial sebum production. This was a split body comparative study in which one group used green tea (GT) topical on one cheek and placebo control on the other (*n* = 11). The second group used topical green tea and lotus (GT-L) on one cheek and placebo control on the other (*n* = 11). The GT topical consisted of 5% green tea and the GT-L formulation contained 2.5% green tea and 2.5% lotus extract. Both groups were instructed to apply their respective topicals at bedtime for 60 days. Sebumeter was used to measure sebum secretion on both cheeks at baseline and days 15, 30, 45, and 60. After 60 days, the GT topical group had a significant reduction in sebum secretion from baseline, compared to placebo control (27% reduction, *p* = 0.006). The GT-L group also had a significant reduction in sebum secretion from baseline to day 60 compared to placebo control (25% reduction, *p* = 0.002). There were no adverse events reported in either GT or GT-L group. This study showed that GT and GT-L topicals were both effective in significantly reducing sebum secretion. Additionally, even though the GT-L combination topical had a smaller percentage of GT extract (2.5% vs. 5% in GT group), GT-L reduced sebum secretion to a higher degree than GT topical alone; this could indicate an additive effect of GT and L extracts. These findings suggest that GT and GT-L combination topicals could be used to treat skin diseases that are associated with increased sebum secretion, such as acne vulgaris. Limitations of this study include a small sample size and that it only included healthy men. Future studies should focus on studying the effects of the two formulations in patients with acne vulgaris.

In a study by Mahmood et al., 2010 [34], ten healthy men age 24–40 years-old applied 3% GT topical to their cheeks for eight weeks. A sebumeter was used to measure the amount of sebum secretion at 1, 2, 3, 4, and 8 weeks which was used to calculate the percent change in sebum production from week 1 to week 8. The results showed that the 3% GT topical led to a significant reduction in sebum production during the 8 week study period (*p* < 0.05). Specifically, sebum production decreased by nearly 10% in the first week and as much as 60% by week 8. No side effects were noted. Limitations of this study include small sample size, no comparison treatment or placebo control, and it did not test individuals with skin diseases known to have high sebum production.

### 3.2. Acne Studies

#### 3.2.1. Topical Tea Polyphenols

Sharquie et al., 2008 [35] conducted a single-blind randomized clinical trial examining 2% tea lotion and 5% zinc sulphate solution in treatment of acne vulgaris. The tea type was not specified. Forty-seven subjects (33 women, 14 men) aged 13–27 years-old were enrolled between June 2006 and December 2007 in Iraq. Subjects were randomly assigned one of two groups: 2% tea lotion (*n* = 24) or 5% zinc sulphate solution (*n* = 23); both groups applied their respective topical twice daily for 2 months. Inflammatory lesion counting was performed every 2 weeks and graded as follows: mild acne (<20 pustules and <10 papules) and moderate acne (20–40 pustules and >30 papules). It was considered that subjects had a “good response” to treatment if the inflammatory lesion count decreased by >50%; “moderate response” when decrease was 10%–50%; and “no response” when lesion count decreased by <10%. The 2% tea lotion group experienced a significant reduction in inflammatory lesion count (papule count decrease with treatment, *p* = 0.0001; pustule count decrease with treatment, *p* = 0.003). Eighty-five percent of subjects had a good or moderate response and 15% had no response to treatment. There was no significant difference in inflammatory lesion count (papule or pustule) in the group treated with 5% zinc sulphate compared to baseline. Adverse event reported by the 2% tea lotion group was mild itching (5 subjects) at the beginning of the treatment period. The 5% zinc sulphate group also reported itching (2 subjects), as well as burning sensation (5 subjects). No serious side effects were reported in either group. One limitation of this study is the small sample size (*n* = 20 in each group that completed the study). Nevertheless, 2% tea lotion is promising for its effect of decreasing inflammatory lesion count. Future studies could be done to compare it to other known topical treatments for acne to further assess its efficacy and safety profile.

A randomly-assigned split body (face) trial was conducted by Yoon et al., 2013 [31] to examine the effectiveness of 1% EGCG and 5% EGCG topicals compared to vehicle containing 3% ethanol in decreasing acne lesion count. Each group applied the assigned topical to one side of the face (1% EGCG or 5% EGCG); both groups applied the vehicle topical to the other side of face. Non-inflammatory and inflammatory lesions were counted. A revised Leeds score, which represents inflammatory and non-inflammatory acne counts, was used as a global assessment of acne severity. The mean Leeds score at baseline was 5.1 ± 0.4. At eight weeks, the mean Leeds score for the 1% EGCG group was 1.2 ± 0.4 and for the 5% EGCG group it was 1.7 ± 0.6. The reduction in Leeds score was significant in both 1% EGCG and 5% EGCG groups (*p* < 0.05). Specifically, non-inflammatory lesions reduced by 79% and inflammatory lesions by 89% after eight weeks of 1% EGCG use compared to baseline (*p* < 0.05). The non-inflammatory and inflammatory lesion reduction was similar in 5% EGCG group (*p* < 0.05), however, the specific lesion counts and percentage improvement were not provided. Overall, this study showed that 1% and 5% EGCG were both effective in improving acne vulgaris without major side effects.

A single-blind randomized controlled study by Sharquie et al., 2006 [36] investigated the effects of 2% tea lotion compared to placebo lotion in its ability to decrease acne lesion count. Sixty subjects, aged 14–22 years-old (35 women, 25 men) participated in the study for a duration of 2 months between October 2002 and October 2004 in Iraq. The treatment group applied 2% tea lotion twice a day for 2 months and inflammatory lesion count (papules and pustules) was done at weeks 4 and 8. The control group underwent the same procedure but used a control lotion, which consisted of distilled water. The 2% tea lotion group had a significant decrease in inflammatory lesion count at 8 weeks post-treatment (baseline papules 12 ± 3.3, week 8 papules 8.1 ± 19, *p* < 0.001; baseline pustules 20.7 ± 5.8, week 8 pustules 8.9 ± 2.3, *p* < 0.001). Eighty-eight percent of subjects in the treatment group reported being fully or partially satisfied with the treatment outcome and 12% were not satisfied. The control group did not have a significant reduction in lesion count at 8 weeks compared to baseline. In this group, 83.3% of subjects were not satisfied with the outcome and 16.7% were partially or fully satisfied. There were no adverse events reported. This study suggests that 2% tea lotion is an effective topical alternative in acne management.

Elsaie et al., 2009 [37] studied the effectiveness of 2% green tea lotion in reducing acne severity. The study had 20 subjects, aged 15–36 years-old (14 women, 6 men) and was conducted in Egypt between May 2007 and February 2008. Acne severity was assessed at baseline via total lesion count (TLC) for pustules and papules and severity index (SI). The TLC at baseline was considered 100% and any decrease during treatment was considered an improvement. Baseline images were taken and subjects were instructed to apply the 2% green tea lotion twice daily for 6 weeks with follow up every 2 weeks. The results showed a mean TLC decrease from 24 (baseline) to 10 after 6 weeks of treatment (58.33% reduction, *p* < 0.0001, 95% confidence interval (CI) = 8.58–19.42). The mean SI also decreased from 2.05 (baseline) to 1.25 after 6 weeks of treatment (39.02% reduction, *p* < 0.0001, 95% CI = 0.54–1.26). Five subjects reported stinging sensation or itching, but these resolved within 2–3 days of treatment. Small sample size and lack of comparison, or control group, were limitations in this study. The true effect of the topical green tea lotion was not clear with the lack of a control treatment. Nevertheless, it showed that 2% green tea lotion could be beneficial in acne treatment by reducing acne lesion count and warrants future controlled studies.

In a study by Jung et al., 2012 [38] conducted in Korea, a green tea extract compound called polyphenon-60 was used to examine its potential therapeutic effects on acne vulgaris. Sixty subjects with mild to moderate acne applied 20 mg/mL polyphenon-60 twice daily for 8 weeks; their acne lesion count was assessed at baseline and week 8. Eight weeks post-treatment with polyphenon-60, there was a 61% decrease in open comedones (*p* < 0.05) and 28% decrease in the number of pustules (*p* < 0.05) compared to baseline. There was no significant decrease in the number of closed comedones at week 8 compared to baseline. No major adverse events were noted. This study did not have a control group or control treatment.

#### 3.2.2. Systemic Tea Polyphenols

Lu et al., 2016 [39] conducted a randomized, double-blind, placebo controlled trial to investigate whether systemic green tea supplementation could improve acne in post-adolescent women. The study enrolled 80 women aged 25–45 years-old with moderate to severe acne, as determined by the Investigators Global Assessment score of 3 or 4 (scale 0–5). The study was conducted in Taiwan between May 2012 and October 2013. One group (*n* = 40) received 1500 mg decaffeinated green tea daily (1 capsule 500 mg GT 30 min after meal, 3 times daily) for four weeks. The second group received cellulose capsules (placebo), which looked identical to GT capsules for blinding purposes. A blinded dermatologist recorded inflammatory and non-inflammatory lesion counts on the forehead, cheeks, nose, perioral area, chin, and entire face at baseline and at four weeks post-treatment. There was a significant decrease in the inflammatory lesion counts on the nose (*p* = 0.03), perioral area (*p* = 0.04), and chin (*p* = 0.03) in the green tea group compared to placebo group. There was no significant difference in lesion counts on forehead, cheek, or in the total lesion count between GT and placebo groups. Within the GT group, subjects had significantly lower inflammatory lesion counts on the forehead (*p* = 0.04) and cheeks (*p* = 0.04), as well as lower total lesion count (*p* = 0.03) at the end of treatment compared to baseline. Within the placebo group, there was significant decrease in inflammatory lesion count on cheeks (*p* = 0.01) and chin (*p* = 0.01), as well as total lesion count (*p* = 0.02). Adverse events included mild constipation and abdominal discomfort (three subjects). Placebo group adverse events included thirst and difficulty falling asleep (two subjects). However, no major side effects were reported. Even though decaffeinated GT treatment resulted in decreased lesion counts on nose, perioral area, and chin compared to placebo, the placebo group itself had significant within group difference in lesion counts before and after treatment. Additional studies with more subjects and longer duration are needed to assess the effectiveness of systemic green tea in treatment of acne.

## 4. Discussion

Polyphenols are naturally occurring compounds found in many common foods and plants, including *Camellia sinensis* [13], the plant that gives rise to a variety of teas. Their natural-based ingredients, cost effectiveness and non-invasive attributes have made them increasingly attractive to patients, compared to prescription drugs [40], and this has led to an increase in polyphenol studies examining their effects on the skin. In this review, we include eight studies focusing on tea polyphenols and their effects on sebum production and acne. All eight studies reported that tea polyphenols were effective at either reducing sebum production or improving acne severity in at least one outcome measure.

### 4.1. Tea Polyphenol Bioavailability

Bioavailability of polyphenols varies depending on the polyphenol forms within the dietary source [41]. EGCG, the most abundant catechin found in tea, is primarily absorbed in the small intestine and metabolized by colonic flora in the large intestine [42]. Studies show that bioavailability varies among catechins where EGCG is readily present in plasma after intake, indicating high bioavailability, whereas galloylated catechins have never been recovered in urine samples [41]. Fasting, fish oils, albumin, soft water, piperine, Vitamin C and storage in cool and dry conditions can increase the oral bioavailability of EGCG [42]. Oral bioavailability is reduced by oxidation with air, metal ions, catechol-O-methyl transferase (COMT) polymorphisms, and gastrointestinal inactivation [43]. Yang et al., 1998 [44] showed that maximal EGCG, EGC, and EC plasma concentration was reached between 1.5 and 2.5 h after consumption of green tea, and they became undetectable in plasma after 24 h. Studies have found extensive variance in bioavailability across polyphenols, including tea polyphenols, which may be driven by the nature of study subject diets and their levels of metabolizing enzymes [41]. Therefore, future studies are needed to further investigate bioavailability of tea polyphenols.

### 4.2. Skin Penetration

The partition coefficients of tea polyphenols are shown in Table 1 [45]. EGC is the most hydrophilic molecule, while ECG is the least polar [45]. A study by dal Belo et al., 2009 [46] investigated application of 6% green tea extract on the human skin obtained from abdominal surgery. The results showed that EGCG was retained within the skin, with higher levels of EGCG detected in the stratum corneum, followed by the epidermis and dermis. This suggests that EGCG is more non-polar as non-polar compounds tend to stay in the stratum corneum rather than get into the epidermis and dermis. Additionally, a study by Zillich et al., 2013 [47] used ex vivo pig skin and showed that both size of the molecule and hydrophobicity of the molecule impact skin permeation of green tea compounds. However, studies on human skin penetration by tea polyphenols are limited in number and further research in this area is warranted.

### 4.3. Tolerability

Overall, studies show that topical and systemic tea polyphenols tend to be well tolerated. A study by Elsaie et al. [37] showed minimal side effects to 2% green tea topical. Ten percent of subjects experienced stinging, which resolved within 48 h, and 15% of subjects experienced local pruritus, which resolved by day three. In a study by Lu et al., 2016 [39], systemic green tea was given to subjects three times daily for four weeks and this regimen was well tolerated. One subject experienced mild constipation and two others experienced mild abdominal pain. Of the eight studies included in this review, none reported major adverse events and the tea polyphenols were generally well tolerated in topical and systemic formulations. Further studies investigating the impact of various dosages should be considered.

### 4.4. Mechanisms of Action

Acne is thought to be due to the activity of bacteria called *Propionibacterium acnes* (*P. acnes*), sebum production by the sebaceous gland, keratinization of the follicular keratinocytes, and inflammation [17]. Various mechanisms of action for the clinical efficacy of tea polyphenols in reducing acne severity have been suggested. *P. acnes* [48], *Propionibacterium granulosum* [49], *Staphylococcus aureus* [49] and *Staphylococcus epidermidis* [49] are bacteria present on the skin of acne patients. A recent study by Li et al., 2015 [50] suggests that the clinical efficacy of green tea polyphenols may be due to their anti-microbial properties against these bacteria. The study compared topical green tea extract (EGCG content of 0.081%), pomegranate juice, and pomegranate extract in terms of their anti-microbial properties. The results showed that 98% of *P. acnes, P. granulosum, S. aureus,* and *S. epidermidis* was inhibited at concentrations of 400 µg gallic acid equivalents (GAE)/mL GT or less.

In addition to the anti-microbial properties of tea polyphenols, other mechanisms have been proposed to explain the improvement in acne from topical green tea administration. Yoon et al., 2013 [31] postulated that this improvement was due to the modulation of the M locus protein kinase-Sterol regulatory element-binding protein 1 (MLPK-SREBP-1) signaling pathway, which leads to reduction in lipogenesis. Additionally, EGCG increased apoptosis of the SEB-1 cell line of sebocytes and led to a reduction in the mean colony forming units of *P. acnes*. Altogether, these findings further support the notion that EGCG has anti-microbial activity against *P. acnes*. EGCG was also found to reduce transcription of the nuclear factor-κB (NF-ĸB) pathway and reduce inflammation. Overall, studies suggest that tea polyphenols exert their effect on sebum production and acne via several mechanisms, including acting as anti-microbial, anti-lipogenic, anti-inflammatory molecules.

### 4.5. Limitations

Interpretation of this review should be considered in light of the limited number of studies that were available. Many of the studies had small sample sizes and some studies had poor research design, including a lack of control group. More studies of high quality are needed to establish the efficacy of tea polyphenols for controlling sebum production, as well as preventing or managing acne vulgaris.

## 5. Conclusions

Our search produced a limited number of results for how tea polyphenols may be used in reducing sebum secretion and acne pathology. While all the studies used some form of tea polyphenols, the exposures were not uniform. Some studies included unknown types of tea and with unspecified amounts of tea polyphenols as exposure. However, based on this review there is some evidence that tea polyphenols used orally and topically may be beneficial for skin health and more specifically, for reducing sebum production by the sebaceous glands and for the prevention and treatment of acne vulgaris. Physicians and other health care professionals should be aware of the studies examining the beneficial effects of tea polyphenols as they could potentially be used as alternatives in skin care. Research studies of high quality and with large sample sizes are needed to assess the efficacy of tea polyphenols in topical and oral prevention of acne vulgaris and lipid synthesis by the sebaceous glands.

## Figures and Tables

**Table 1:** Partition coefficients of green tea polyphenols.

Polyphenol	K_(non-polar/polar)_
Epicatechin gallate (ECG)	6.25
Epigallocatechin gallate (EGCG)	2.94
Epicatechin (EC)	2.38
Catechin (C)	2.33
Epigallocatechin (EGC)	0.93

## Appendix A

**Table A1:** Tea polyphenol studies—summary.

Tea	Polyphenol Subtypes and Content	Therapy (Topical or Systemic; Dosage)	Comparison (or Control/Placebo)	Subjects (*n*, Age)	Study Design, Duration	Major Outcome Measures	Major Results	Adverse Events	Reference
Sebum Production Studies									
Green tea	GT formulation: 5% green tea; GT-L formulation: 2.5% green tea + 2.5% lotus extract.	Green tea (GT) Topical	Green tea + Lotus (GT-L) Topical; Placebo Topical	*n* = 22; all healthy men; Age 22–28 years-old.	Single-blinded, placebo-controlled, monocentric study; Pakistan (months December-February); 2 groups (G): G1: used GT topical on one cheek, placebo on the other; *n* = 11; G2: GT-L topical on one cheek, placebo on the other; *n* = 11. Intervention length: 60 days; Amount of tea polyphenol: information not provided; Frequency of application: once daily at bedtime; Follow up time: baseline, day 15, 30, 45, 60; Sebum excretion on both cheeks measured with Sebumeter at each visit.	Percent change in sebum secretion every 15 days (calculated by formula % change = ((D_x_ − D_0_)/D_0_) × 100; D_x_ = sebum value on day 15, 30 45, 60; D_0_ = baseline sebum value)	G1 (GT group): significant reduction in sebum secretion from baseline to 60 days of treatment compared to placebo control (*p* = 0.0060) (27% decrease from baseline); G2 (GT-L group): significant reduction in sebum secretion from baseline to 60 days of treatment compared to placebo control (*p* = 0.0002) (25% decrease from baseline); Placebo: no significant effect on reducing sebum secretion.	None reported	Mahmood et al., 2013 [15]
Green tea	3% GT extract	Topical	No control group	*n* = 10; Age 24 to 40 years-old; all healthy men	Therapeutic study Intervention length: 60 days; Frequency of topical use: not stated; Amount of topical: not stated; Follow up: skin sebum production measured at week 1, 2, 3, 4, 6, and 8 via sebumeter.	Percent change in skin sebum production (calculated via formula % change = ((A − B)/B) × 100 A = individual value of any parameter (from 1st to 8th week); B = zero value for that parameter.	3% GTE formulation decreased sebum production by nearly 10% at week 1, and 60% at week 8) (*p* < 0.05)		Mahmood et al., 2010 [34]
Acne Studies									
Green tea	Amount of tea polyphenols: Each GTE capsule contained: 285.6 mg EGCG, 78.7 mg ECG, 38.5 mg EGC, 24 mg EC, 21.25 mg GCG, <0.3 mg GC, <0.3 mg caffeine, 51.65 mg cellulose	Systemic Decaffeinated GTE; each capsule 500 mg decaffeinated GTE or cellulose	Placebo (cellulose)	*n* = 80; All women; Age 25–45 years-old with moderate to severe acne (determined by Investigator’s Global Assessment (score of 3 or 4 on a scale 0 to 5)); None of the women received systemic retinoid or hormone treatment during previous 3 months.	Randomized, double blind, placebo controlled trial; May 2012 to October 2013, Taiwan; Intervention length: 4 weeks; Follow up: baseline and at 4 weeks; G1: received decaffeinated GTE for 4 weeks *n* = 40; G2: placebo (pure cellulose) for 4 weeks *n* = 40; Products packaged into identical capsules for blinding purposes; Frequency: Subjects took 1 capsule 30 min after meals 3 times daily for 4 weeks.	Acne lesion counts (inflammatory and non-inflammatory) recorded for entire face by blinded dermatologist at baseline and at 4 weeks	Between group comparison: G1 vs. G2: significant reduction in inflammatory lesion counts on nose (*p* = 0.03), perioral area (*p* = 0.04) and chin (*p* = 0.03); no difference in total lesion count; G1 did not have significant reduction on forehead, cheek and total lesion count compared to placebo; Within group comparison: G1: Compared with baseline, G1 (decaffeinated GTE) had significantly lower number of inflammatory lesions on forehead (*p* = 0.04), cheeks (*p* = 0.04) and entire face (total lesion count) (*p* = 0.003); G2: in placebo group, there was significant reduction in inflammatory lesion count on cheeks (*p* = 0.01), chin (*p* = 0.01) and entire face (*p* = 0.02).	G1: 1 subject developed mild constipation, 2 subjects abdominal discomfort after GTE treatment; G2: 1 subject thirsty, 1 difficulty falling asleep; No major adverse effects noted.	Lu et al., 2016 [39]
Tea	Apple brand tea leaves; Amount of tea polyphenol: not stated	Topical 2% tea lotion;	5% zinc sulphate solution	*n* = 47; 33 females; 14 males; Age 13–27 years-old with acne vulgaris (severe acne excluded); Excluded comedonal, severe acne and nodulo-cystic acne and if on acne treatment in past 2 months; also systemic disease therapy with ie corticosteroids, hormones, medications that induce acniform rash.	Single-blind randomly comparative therapeutic clinical trial; Iraq, June 2006 to December 2007; Intervention length 8 weeks; Frequency of topical: twice daily; G1: 2% tea lotion *n* = 24 (17 females, 7 males); G2: 5% zinc sulphate solution *n* = 23 (16 females, 7 males); Follow up: Inflammatory (papules and pustules) lesions counted every 2 weeks for a total of 8 weeks; Photograph taken at baseline and subsequent visits.	Clinical improvement of acne vulgaris—scored by inflammatory lesion counting before and after treatment: Mild acne: <20 pustules and <10 papules; Moderate acne: 20–40 pustules, >30 papules). Good response: inflammatory (papules and pustules) lesion count decrease by >50%; Moderate response: lesion count decrease between 10% and 50%; No response: reduction in lesion count <10%.	*n* = 40 completed the study; *n* = 20 in each group; G1: 2% tea lotion statistically significant decrease in number of inflammatory lesions; Response to treatment: 15%—no response; 25%—moderate; 60%—good. Papule count before treatment 23.3 ± 10.9; After treatment 12 ± 8.7, statistically significant difference (*p* = 0.0001). Pustule count before treatment 29.5 ± 5.8; After treatment 14.5 ± 10.7, statistically significant reduction (*p* < 0.001). G2: zinc sulphate beneficial but result not statistically significant Response to treatment: 35%—no response; 50%—moderate; 15%—good. Papules before treatment 19.5 ± 9.7 and after treatment 14.4 ± 8.6 (reduction not statistically significant; *p* = 0.08); Pustules before treatment 19.4 ± 9.3 and after treatment 15.2 ± 9.5 (reduction not significant; *p* = 0.16).	G1: 5 subjects (25%): mild itching only in early course of treatment which then disappeared; G2: burning sensation in 5 subjects (25%), itching in 2 (10%) in early course of treatment which was subsequently reduced; No major adverse effects.	Sharquie et al., 2008 [35]
Green tea	EGCG	1% EGCG and 5% EGCG Topical	Vehicle with 3% ethanol	*n* = 35; 17 men; 18 women; mean age: 22.1 (range not stated).	Randomly assigned split body trial; Intervention length: 8 weeks; Follow up: baseline, week 1, 2, 4, 6, and 8; Frequency of application: twice daily; Amount per application: not stated; G1: used 1% EGCG, *n* = 17; G2: used 5% EGCG, *n* = 18; Both groups applied lotion to one side of face, vehicle containing 3% ethanol to the other.	Dose response relationship between 1% and 5% EGCG in improving acne lesion count; Leeds grading and acne lesion count to calculate changes in non inflammatory lesions (whiteheads, blackheads, comedones) and inflammatory lesions (papules, pustules, nodules) during EGCG application period.	Baseline Leeds score: 5.1 ± 0.4; Week 8 Leeds score: 1.2 ± 0.4 (1% EGCG); 1.7 ± 0.6 (5% EGCG); 1% EGCG resulted in: 79% reduction of non-inflammatory acne (53.8 ± 19.8 mean lesions at baseline, 15.6 ± 6.2 at week 8, *p* < 0.05); 89% reduction in inflammatory acne (10 ± 3.1 mean lesions at baseline, 1.1±0.5 at week 8, *p* < 0.05); 5% EGCG significantly improved inflammatory and non-inflammatory lesion counts (*p* < 0.05), but details not provided.		Yoon et al., 2013 [31]
Tea	Swan brand tea	Topical 2% tea lotion: (prepared by adding 75 mL tea extract + 25 mL ethanol (95% purity))	Control lotion: (prepared by adding 75 mL distilled water + 25 mL ethanol (95% purity))	*n* = 60; 25 males; 35 females; Age 14–22 years-old.	Iraq, October 2002 to October 2004; Intervention length: 2 months; Follow up: at 1 and 2 months; Frequency of topical application: twice daily; Amount applied: not stated; 2 groups: G1: *n* = 30, mean age 17.5 ± 3.9 years, treated with 2% tea lotion twice per day for 2 months, clinical evaluation monthly (inflammatory lesion count—papules and pustules). G2: *n* = 30, mean age 18.1 ± 3.7 years treated in the same manner but with control lotion.	Inflammatory lesion count (papules and pustules) assessed as follows: Mild acne: < 20 pustules, papules < 10; Moderate acne: 20–40 pustules, 10–30 papules; Severe acne: pustules > 40, papules > 30. Response to treatment classified as: Good response: >50% reduction in inflammatory lesion count; Moderate response: 10%–50% reduction in inflammatory lesion count; No response: <10% reduction in inflammatory lesion count. Patient satisfaction to treatment assessed as: - full satisfaction - partial satisfaction - no satisfaction	*n* = 49 completed treatment; G1: *n* = 25 Significant reduction in lesion count of papules (baseline 12 ± 3.3, week eight 8.1 ± 0.19, *p* < 0.001) and pustules (baseline 20.7 ±5.8, week eight 8.9 ± 2.3, *p* < 0.001); G2: *n* = 24. No significant reduction of papules or pustules at 8 weeks compared to baseline. Patient satisfaction after 8 weeks: G1: 64% fully satisfied, 6 patients (24%) partially satisfied, 12% not satisfied; G2: 83.3% not satisfied, 12.5% partially satisfied, 4.2% fully satisfied.	None	Sharquie et al., 2006 [36]
Green tea		Topical 2% green tea lotion (prepared by adding 75 mL tea extract + 25 mL ethanol (95% purity)		*n* = 20; 6 males; 14 females; Age 15–36 years old.	Egypt, May 2007 to February 2008; Intervention length: 6 weeks; Follow up: every 2 weeks; Frequency of application: twice daily; Amount of topical applied: not stated; Acne severity assessed, baseline photographs taken, green tea lotion given to be applied on face twice daily for 6 weeks, follow up every 2 weeks; TLC considered 100% at baseline and any decrease was considered improvement.	Change in TLC and SI scores at the end of treatment compared to baseline; Acne severity assessed every 2 weeks via total lesion count (TLC) and severity index (SI); TLC = papules + pustules; SI was determined as follows: SI = 1 if TLC < 10 SI = 2 if TLC = 10–20 SI = 3 if TLC > 20	Mean TLC decreased from 24 (baseline) to 10 after 6 weeks of treatment (58.33% reduction, *p* < 0.0001, 95% CI = 8.58–19.42); Mean SI decreased from 2.05 (baseline) to 1.25 after 6 weeks of treatment (39.02% reduction, *p* < 0.0001, 95% CI = 0.54–1.26).	Stinging sensation in 2 subjects (10%) on first day of lotion use, disappeared in 48 h; Minimal pruritus in 3 of 20 subjects (15%) on first day of lotion use and lasted 3 days.	Elsaie et al., 2009 [37]
Green tea	Polyphenon-60 (green tea extract compound)	Topical 20 mg/mL polyphenon-60 applied to face twice daily for 8 weeks	None	*n* = 30, with mild to moderate acne; Age: not stated; Proportion of sex: not stated.	Therapeutic study; Intervention length: 8 weeks; Follow up time: baseline and at 8 weeks; Frequency of application: twice daily; Amount applied: not stated; Korea Topical polyphenon-60 (20 mg/mL) from green tea lotion applied onto facial acne twice daily for 8 weeks; Acne lesion count at baseline and at 8 weeks done by dermatologists.	Acne lesion count: open comedones, closed comedones, and pustules at baseline vs. 8 weeks after treatment	8 weeks post-treatment compared to baseline: Number of open comedones decreased by 61% (*p* < 0.05); Number of pustules decreased by 28% (*p* < 0.05); Number of closed comedones was not significantly decreased.		Jung et al., 2012 [38]

Abbreviations: EC: epicatechin; ECG: epicatechin gallate; EGC: epigallocatechin; G: group; G1: group 1; G2: group 2; GC: gallocatechin; GCG: gallocatechin gallate; GT-L: green tea-lotus combination; GT: green tea; GTE: green tea extract; SD: standard deviation; SI: severity index; TLC: total lesion count.

## References

[1] Watson R.R., Preedy V.R., Zibadi S. (2013). Polyphenols in Human Health and Disease.

[2] Habauzit V., Morand C. (2012). Evidence for a protective effect of polyphenols-containing foods on cardiovascular health: An update for clinicians. Ther. Adv. Chronic Dis..

[3] Zern T.L., Fernandez M.L. (2005). Cardioprotective effects of dietary polyphenols. J. Nutr..

[4] Johnston C. (2009). Functional foods as modifiers of cardiovascular disease. Am. J. Lifestyle Med..

[5] Curin Y., Andriantsitohaina R. (2005). Polyphenols as potential therapeutical agents against cardiovascular diseases. Pharmacol. Rep..

[6] Tsao R. (2010). Chemistry and biochemistry of dietary polyphenols. Nutrients.

[7] Sies H., Stahl W. (2004). Nutritional protection against skin damage from sunlight. Annu. Rev. Nutr..

[8] Lambert J.D. (2013). Does tea prevent cancer? Evidence from laboratory and human intervention studies. Am. J. Clin. Nutr..

[9] Afaq F., Syed D.N., Malik A., Hadi N., Sarfaraz S., Kweon M.H., Khan N., Zaid M.A., Mukhtar H. (2007). Delphinidin, an anthocyanidin in pigmented fruits and vegetables, protects human HaCaT keratinocytes and mouse skin against UVB-mediated oxidative stress and apoptosis. J. Investig. Dermatol..

[10] Hwang Y.P., Oh K.N., Yun H.J., Jeong H.G. (2011). The flavonoids apigenin and luteolin suppress ultraviolet A-induced matrix metalloproteinase-1 expression via MAPKs and AP-1-dependent signaling in HaCaT cells. J. Dermatol. Sci..

[11] Heinrich U., Moore C.E., de Spirt S., Tronnier H., Stahl W. (2011). Green tea polyphenols provide photoprotection, increase microcirculation, and modulate skin properties of women. J. Nutr..

[12] OyetakinWhite P., Tribout H., Baron E. (2012). Protective mechanisms of green tea polyphenols in skin. Oxid. Med. Cell. Longev..

[13] Graham H.N. (1992). Green tea composition, consumption, and polyphenol chemistry. Prev. Med..

[14] Smith K.R., Thiboutot D.M. (2008). Thematic review series: Skin lipids. Sebaceous gland lipids: Friend or foe?. J. Lipid Res..

[15] Mahmood T., Akhtar N., Moldovan C. (2013). A comparison of the effects of topical green tea and lotus on facial sebum control in healthy humans. Hippokratia.

[16] Layton A.M. (2001). Optimal management of acne to prevent scarring and psychological sequelae. Am. J. Clin. Dermatol..

[17] Zouboulis C.C., Eady A., Philpott M., Goldsmith L.A., Orfanos C., Cunliffe W.C., Rosenfield R. (2005). What is the pathogenesis of acne?. Exp. Dermatol..

[18] Melnik B.C., Zouboulis C.C. (2013). Potential role of FoxO1 and mTORC1 in the pathogenesis of western diet-induced acne. Exp. Dermatol..

[19] Monfrecola G., Lembo S., Caiazzo G., de Vita V., di Caprio R., Balato A., Fabbrocini G. (2016). Mechanistic target of rapamycin (mTOR) expression is increased in acne patients’ skin. Exp. Dermatol..

[20] Agamia N.F., Abdallah D.M., Sorour O., Mourad B., Younan D.N. (2016). Skin expression of mammalian target of rapamycin and forkhead box transcription factor O1, and serum insulin-like growth factor-1 in patients with acne vulgaris and their relationship with diet. Br. J. Dermatol..

[21] Smith T.M., Gilliland K., Clawson G.A., Thiboutot D. (2008). IGF-1 induces SREBP-1 expression and lipogenesis in SEB-1 sebocytes via activation of the phosphoinositide 3-kinase/Akt pathway. J. Investig. Dermatol..

[22] Melnik B.C. (2013). Western diet-mediated mTORC1-signaling in acne, psoriasis, atopic dermatitis, and related diseases of civilization: Therapeutic role of plant-derived natural mTORC1 inhibitors. Bioactive Dietary Factors and Plant Extracts in Dermatology.

[23] Im M., Kim S.Y., Sohn K.C., Choi D.K., Lee Y., Seo Y.J., Kim C.D., Hwang Y.L., Zouboulis C.C., Lee J.H. (2012). Epigallocatechin-3-gallate suppresses IGF-I-induced lipogenesis and cytokine expression in SZ95 sebocytes. J. Investig. Dermatol..

[24] Van Aller G.S., Carson J.D., Tang W., Peng H., Zhao L., Copeland R.A., Tummino P.J., Luo L. (2011). Epigallocatechin gallate (EGCG), a major component of green tea, is a dual phosphoinositide-3-kinase/mTOR inhibitor. Biochem. Biophys. Res. Commun..

[25] Melnik B.C. (2015). Linking diet to acne metabolomics, inflammation, and comedogenesis: An update. Clin. Cosmet. Investig. Dermatol..

[26] Melnik B.C., Schmitz G. (2013). Are therapeutic effects of antiacne agents mediated by activation of FoxO1 and inhibition of mTORC1?. Exp. Dermatol..

[27] Kuo K.L., Weng M.S., Chiang C.T., Tsai Y.J., Lin-Shiau S.Y., Lin J.K. (2005). Comparative Studies on the Hypolipidemic and Growth Suppressive Effects of Oolong, Black, Pu-Erh, and Green Tea Leaves in Rats. J. Agric. Food Chem..

[28] Malahlela P., Motswaledi M.H. (2013). Management of mild to moderate acne vulgaris. S. Afr. Fam. Pract..

[29] Lucky A.W., Barber B.L., Girman C.J., Williams J., Ratterman J., Waldstreicher J. (1996). A multirater validation study to assess the reliability of acne lesion counting. J. Am. Acad. Dermatol..

[30] Doshi A., Zaheer A., Stiller M.J. (1997). A comparison of current acne grading systems and proposal of a novel system. Int. J. Dermatol..

[31] Yoon J.Y., Kwon H.H., Min S.U., Thiboutot D.M., Suh D.H. (2013). Epigallocatechin-3-gallate improves acne in humans by modulating intracellular molecular targets and inhibiting *P. Acnes*. J. Investig. Dermatol..

[32] Leyden J.J. (1997). Therapy for acne vulgaris. N. Engl. J. Med..

[33] Muthu M., Gopal J., Min S.X., Chun S. (2016). Green tea versus traditional Korean teas: Antibacterial/antifungal or both?. Appl. Biochem. Biotechnol..

[34] Mahmood T., Akhtar N., Khan B.A., Khan H.M., Saeed T. (2010). Outcomes of 3% green tea emulsion on skin sebum production in male volunteers. Bosn. J. Basic Med. Sci..

[35] Sharquie K.E., Noaimi A.A., Al-Salih M.M. (2008). Topical therapy of acne vulgaris using 2% tea lotion in comparison with 5% zinc sulphate solution. Saudi Med. J..

[36] Sharquie K.E., Al-Turfi I.A., Al-Shimary W.M. (2006). Treatment of acne vulgaris with 2% topical tea lotion. Saudi Med. J..

[37] Elsaie M.L., Abdelhamid M.F., Elsaaiee L.T., Emam H.M. (2009). The efficacy of topical 2% green tea lotion in mild-to-moderate acne vulgaris. J. Drugs Dermatol..

[38] Jung M.K., Ha S., Son J.A., Song J.H., Houh Y., Cho E., Chun J.H., Yoon S.R., Yang Y., Bang S.I. (2012). Polyphenon-60 displays a therapeutic effect on acne by suppression of TLR2 and IL-8 expression via down-regulating the ERK1/2 pathway. Arch. Dermatol. Res..

[39] Lu P.H., Hsu C.H. (2016). Does supplementation with green tea extract improve acne in post-adolescent women? A randomized, double-blind, and placebo-controlled clinical trial. Complement. Ther. Med..

[40] Levin J., Momin S.B. (2010). How much do we really know about our favorite cosmeceutical ingredients?. J. Clin. Aesthet. Dermatol..

[41] Manach C., Williamson G., Morand C., Scalbert A., Remesy C. (2005). Bioavailability and bioefficacy of polyphenols in humans. I. Review of 97 bioavailability studies. Am. J. Clin. Nutr..

[42] Mereles D., Hunstein W. (2011). Epigallocatechin-3-gallate (EGCG) for clinical trials: More pitfalls than promises?. Int. J. Mol. Sci..

[43] Peter B., Bosze S., Horvath R. (2016). Biophysical characteristics of proteins and living cells exposed to the green tea polyphenol epigallocatechin-3-gallate (EGCG): Review of recent advances from molecular mechanisms to nanomedicine and clinical trials. Eur. Biophys. J..

[44] Yang C.S., Chen L., Lee M.J., Balentine D., Kuo M.C., Schantz S.P. (1998). Blood and urine levels of tea catechins after ingestion of different amounts of green tea by human volunteers. Cancer Epidemiol. Biomarkers Prev..

[45] Yanagida A., Shoji A., Shibusawa Y., Shindo H., Tagashira M., Ikeda M., Ito Y. (2006). Analytical separation of tea catechins and food-related polyphenols by high-speed counter-current chromatography. J. Chromatogr. A.

[46] Dal Belo S.E., Gaspar L.R., Maia Campos P.M., Marty J.P. (2009). Skin penetration of epigallocatechin-3-gallate and quercetin from green tea and Ginkgo biloba extracts vehiculated in cosmetic formulations. Skin Pharmacol. Physiol..

[47] Zillich O.V., Schweiggert-Weisz U., Hasenkopf K., Eisner P., Kerscher M. (2013). Release and in vitro skin permeation of polyphenols from cosmetic emulsions. Int. J. Cosmet. Sci..

[48] Bojar R.A., Holland K.T. (2004). Acne and propionibacterium acnes. Clin. Dermatol..

[49] Bek-Thomsen M., Lomholt H.B., Kilian M. (2008). Acne is not associated with yet-uncultured bacteria. J. Clin. Microbiol..

[50] Li Z., Summanen P.H., Downes J., Corbett K., Komoriya T., Henning S.M., Kim J., Finegold S.M. (2015). Antimicrobial activity of pomegranate and green tea extract on propionibacterium acnes, propionibacterium granulosum, staphylococcus aureus and staphylococcus epidermidis. J. Drugs Dermatol..

